# Samsoeum, a traditional herbal medicine, elicits apoptotic and autophagic cell death by inhibiting Akt/mTOR and activating the JNK pathway in cancer cells

**DOI:** 10.1186/1472-6882-13-233

**Published:** 2013-09-23

**Authors:** Aeyung Kim, Nam-Hui Yim, Jin Yeul Ma

**Affiliations:** 1 Korean Medicine (KM)-Based Herbal Drug Research Group, Korea Institute of Oriental Medicine (KIOM), 483 Expo-ro, Yuseong-gu, Daejeon 305-811, Republic of Korea

**Keywords:** Samsoeum, Apoptosis, Autophagy, Cell cycle arrest, Akt/mTOR, JNK

## Abstract

**Background:**

Samsoeum (SSE), a traditional herbal formula, has been widely used to treat cough, fever, congestion, and emesis for centuries. Recent studies have demonstrated that SSE retains potent pharmacological efficiency in anti-allergic and anti-inflammatory reactions. However, the anti-cancer activity of SSE and its underlying mechanisms have not been studied. Thus, the present study was designed to determine the effect of SSE on cell death and elucidate its detailed mechanism.

**Methods:**

Following SSE treatment, cell growth and cell death were measured using an MTT assay and trypan blue exclusion assay, respectively. Cell cycle arrest and YO-PRO-1 uptake were assayed using flow cytometry, and LC3 redistribution was observed using confocal microscope. The mechanisms of anti-cancer effect of SSE were investigated through western blot analysis.

**Results:**

We initially found that SSE caused dose- and time-dependent cell death in cancer cells but not in normal primary hepatocytes. In addition, during early SSE treatment (6–12 h), cells were arrested in G_2_/M phase concomitant with up-regulation of p21 and p27 and down-regulation of cyclin D1 and cyclin B1, followed by an increase in apoptotic YO-PRO-1 (+) cells. SSE also induced autophagy via up-regulation of Beclin-1 expression, conversion of microtubule-associated protein light chain 3 (LC3) I to LC3-II, and re-distribution of LC3, indicating autophagosome formation. Moreover, the level of B-cell lymphoma 2 (Bcl-2), which is critical for cross-talk between apoptosis and autophagy, was significantly reduced in SSE-treated cells. Phosphorylation of adenosine monophosphate-activated protein kinase (AMPK) was increased, followed by suppression of the protein kinase B/mammalian target of rapamycin (Akt/mTOR) pathway, and phosphorylation of mitogen-activated protein kinases (MAPKs) in response to SSE treatment. In particular, among MAPKs inhibitors, only the c-Jun N-terminal kinase (JNK)-specific inhibitor SP600125 nearly blocked SSE-induced increases in Beclin-1, LC3-II, and Bax expression and decreases in Bcl-2 expression, indicating that JNK activation plays critical role in cell death caused by SSE.

**Conclusions:**

These findings suggest that SSE efficiently induces cancer cell death via apoptosis as well as autophagy through modification of the Akt/mTOR and JNK signaling pathways. SSE may be as a potent traditional herbal medicine for treating malignancies.

## Background

In cancer cells, the balance of cell death with survival is frequently disturbed by the mutation of oncogenes or tumor suppressor genes and by the alteration of signaling pathways [1]. Because perturbation of the cell death process is closely related to cancer progression and resistance to chemotherapy or radiotherapy, accumulating evidence has shown that agents targeting the programmed cell death (PCD) pathway without affecting normal cells play crucial roles as potential drug targets in cancer treatment [2]. PCD, a cell suicide program, plays pivotal roles in the development, tissue homeostasis, and elimination of damaged cells as a basic biological phenomenon and can be classified according to morphological differences as apoptosis (type I), autophagy (type II), and programmed necrosis (type III) [3,4].

Apoptosis, a major type of cell death that occurs when DNA damage is unrecoverable, is characterized by distinct morphological and biochemical changes such as cell shrinkage, membrane blebbing, chromatin condensation, and ultimately, fragmentation of cells into membrane-enclosed vesicles designated as apoptotic bodies, loss of adherence to extracellular matrix, activation of proteases, and externalization of phosphatidylserine [5]. Autophagy is a physiological process that provides energy required for the turnover of cellular proteins and other macromolecules under certain stress conditions such as nutrient deprivation, hypoxia, and metabolic, genotoxic, and oxidative stress. Autophagy is regarded as a cell survival and protection mechanism; thus, it may play a negative role in cancer therapy and limit the therapeutic efficacy of chemotherapeutic agents [6]. However, recent studies have reported that excessive and sustained autophagy by various anti-cancer therapies can eventually induce cell death in many kinds of cancer cells [7-11], supporting the notion that autophagy may act as either a guardian or an executor. In addition, especially in apoptosis-defective cells, autophagy triggered by cytotoxic drugs promotes cancer cell death via excessive engulfment of cytoplasmic cellular components within a vacuole (autophagosome) and delivery to the lysosome for degradation [12]. In some circumstances, autophagy and apoptosis occur simultaneously in cancers and may be interconnected by certain upstream signaling pathways [13,14]. Among them, the protein kinase B/mammalian target of rapamycin/p70S6K (Akt/mTOR/p70S6K) signaling pathway is coordinately regulated in both apoptosis and autophagy, and Beclin-1 is an integrator that regulates apoptotic and autophagic activities [15].

The autophagy gene Beclin-1, as part of the class III phosphatidylinositol 3 kinase (PI3K) complexes, participates in the formation of autophagic vesicles and is critical in the mediation of localization of other autophagic proteins to pre-autophagosomal membranes. Overexpression of Beclin-1 in human cervical cancer cells has been reported to induce massive autophagic cell death and inhibit cancer cell growth [16]. Some anti-apoptotic B-cell lymphoma 2 (Bcl-2) family members including Bcl-2 and Bcl-xL can interact with Beclin-1, and the Beclin-1/Bcl-2 complex functions as an inhibitor of autophagy-induced cell death. Thus, dissociation of Beclin-1 from Bcl-2 is required for Beclin-1-dependent autophagy induction [17]. Similarly, the constitutively activated class I PI3K/Akt pathway inhibits both apoptosis and autophagy because it acts as a positive regulator of the mTOR signaling pathway and plays a crucial role in cancer cell survival. Thus, disruption of the class I PI3K/Akt pathway by anti-cancer agents induces autophagy [18,19].

Samsoeum (SSE, *Shensuyin* in Chinese, *Jinsoin* in Japanese), a traditional herbal medicine, was first described during the Song Dynasty of China and has been widely used as a remedy for headache, cough, rhinorrhea, and fever. SSE also has been used to treat congestion with phlegm, tidal fever, and emesis. Recent studies have reported the pharmacological efficacy of SSE in allergic and asthma reactions and pulmonary damage from ozone [20]. SSE modulates allergic and inflammatory reactions via inhibition of the expression of cyclooxygenase 2 (COX-2) and inflammatory cytokines and suppression of nuclear factor-κB (NF-κB) activation [21]. However, the anti-cancer effect of SSE and its exact mechanism of action remain to be examined. Therefore, the present study aimed to elucidate the effect of SSE on the cell growth and cell death in cancer cells and investigate the detailed mechanism of its anti-cancer activity.

## Methods

### Cell lines

The human gastric carcinoma AGS cell line, human fibrosarcoma HT1080 cell line, human epidermoid carcinoma A431 cell line, and murine melanoma B16F10 cell line were purchased from American Type Culture Collection (ATCC, Manassas, VA). Each cell line was maintained as a monolayer culture in Roswell Park Memorial Institute (RPMI) 1640 or Dulbecco’s Modified Eagle Medium (DMEM; Lonza, Walkersville, MD) supplemented with 10% (v/v) heat-inactivated fetal bovine serum (FBS; GIBCO/Invitrogen, Carlsbad, CA), 100 units/mL penicillin, and 100 μg/mL streptomycin (Welgene) at 37°C in a humidified 5% CO_2_ incubator. Murine hepatocytes were isolated from 6–8 weeks old female ICR mouse purchased from Nara Bio animal center (Nara Biotech, Korea). Mice were housed under standard conditions at a temperature of 24 ± 1°C and humidity of 55 ± 5%, and experimental procedures were approved by Korea Institute of Oriental Medicine Care and Use Committee with a reference number 12–122. Mice were cared for in accordance with the dictates of the National Animal Welfare Law of Korea and experiments were carried out in accordance with the Korea Institute of Oriental Medicine Care Committee Guidelines. Murine hepatocytes were isolated using a perfusion system with some modification [22]. After suspending in the William’s E medium containing 10% FBS, 100 IU/mL insulin, 2 mM L-glutamine, 15 mM HEPES, 100 units/mL penicillin, and 100 μg/mL streptomycin, hepatocytes were seeded on the culture plate coated with 10% gelatin/phosphate buffered saline (PBS), and incubated at 37°C in a humidified 5% CO_2_ incubator.

### Antibodies and reagents

Propidium iodide (PI), Ribonuclease A (RNase A) from bovine pancreas, and 3-(4,5-Dimethyl-2-thiazolyl)-2,5-diphenyltetrazolium bromide (MTT) were purchased from Sigma Chemical Co. (St Louis, MO, USA). Antibodies against Cyclin D1, Cyclin B1, Cdc25, and α-tubulin were obtained from Santa Cruz Biotechnology Inc. (Santa Cruz, CA, USA). Anti-p21^Waf1/Cip1^, anti-p27^Kip1^, anti-caspase-3, poly (ADP-ribose) polymerase (PARP), anti-p38, anti-phospho-p38 (Thr180/Tyr182), anti-extracellular signal-related kinase1/2 (ERK), anti-phospho-ERK (Thr202/Tyr204), anti-c-Jun-N-terminal kinase (JNK), anti-phopsho-JNK (Thr183/Tyr185), anti-Akt, anti-phopho-Akt (Ser473), anti-mTOR, anti-phospho-mTOR (Ser2481), anti-adenosine monophosphate activated-activated protein kinase (AMPK), anti-phospho-AMPK (Thr172), anti-Bcl-2, anti-Bax, and anti-Beclin-1 antibodies were purchased from Cell Signaling Technology (Danvers, MA, USA). Anti-microtubule-associated protein light chain 3 (LC3) and anti-cleaved caspase-3 antibodies were from Sigma Chemical Co. and Abcam (Cambridge, UK), respectively. All of the other chemicals and solvents used were analytical grade.

### Preparation of herbal extract, Samsoeum (SSE)

Samsoeum (SSE) is composed of 12 Korean medicinal herbs (Table 1) which were obtained from Yeongcheon Oriental Herbal Market (Yeongcheon, Korea). Identification of all herbs was confirmed by Prof. Ki Hwan Bae of the College of Pharmacy, Chungnam National University (Daejeon, Korea), and all voucher specimens were deposited in the herbal band in Korea Institute of Oriental Medicine (KIOM, Daejeon, Korea). A decoction of SSE was extracted in distilled water by heating for 3 h at 115°C in an extractor (Cosmos-600 Extractor, Gyeonseo Co., Inchon, Korea), filtered using standard testing sieves (150 μm, Retsch, Haan, Germany), and then concentrated to dryness in a lyophilizer. The freeze-dried SSE extract was dissolved in distilled water at concentration of 25 mg/mL, filtered through a 0.22 μm disk filter, and then kept at 4°C prior to use.

**Table 1:** The prescription of Samsoeum (SSE)

**Herbal name**	**Dose (g)**	**Relative amount (%)**
Paerillae Folium	3.75	9.1
Puerariae Radix	3.75	9.1
Pinelliae Tuber	3.75	9.1
Angelicae Decursivae Radix	3.75	9.1
Ginseng Radix	3.75	9.1
Poria Sclerotium	3.75	9.1
Autantii Fructus Immaturus	2.81	6.8
Platycodonis Radix	2.81	6.8
Glycyrrhizae Radix et Rhizoma	2.81	6.8
Citri Unshius Pericarpium	2.81	6.8
Zingiberis Rhizoma Crudus	3.75	9.1
Zizyphi Fructus	3.75	9.1
**Total**	**41.24**	**100**

### Cell viability and cell death assay

Cells were seeded at a density of 5 × 10^3^ cells/well in 96-well culture plates, and then incubated with concentrations of SSE between 10 to 250 μg/mL. Untreated ‘control’ cells were incubated with DMSO at final concentration of 0.01%. After 24 h of treatment, cells were incubated with 10 μL of MTT solution (5 mg/mL in PBS) for additional 4 h, formazan precipitates were dissolved by dimethyl sulfoxide (DMSO) and then absorbance was measured at 570 nm with Infinite® M200 microplate reader (TECAN Group Ltd. Switzerland). For cell death evaluation, SSE-treated cells were stained in 0.4% trypan blue solution (GIBCO) and then counted using a hemacytometer (Neubauer Improved, Marienfeld, Germany) under inverted microscope (Olympus CKX41SF; Olympus Optical Co. LTD, Tokyo, Japan). In the experiment with inhibitors, cells were treated with indicated concentrations of SSE for 24 h with or without a 1 h pretreatment with 10 μM SP600125 (Calbiochem, San Diego, CA), 10 μM SB203580 (Calbiochem), 10 μM PD98059 (Calbiochem), 100 μM 3-methyladenine (3-MA; Sigma), or 10 μM z-VAD-fmk (Calbiochem).

### Cell cycle analysis

Cells were seeded on 60 mm culture dishes at a density of 5 × 10^5^ cells/dish and allowed to adhere overnight. After incubation with 50 μg/mL of SSE for 6, 12, and 24 h, cells were harvested, washed twice with PBS, and fixed with ice-cold 70% ethanol at −20°C for 24 h. Subsequently, cells were centrifuged, washed once with PBS, and then intracellular DNA was labeled with 0.5 mL of cold propidium iodide (PI) solution (0.1% Triton X-100, 0.1 mM EDTA, 50 μg/mL RNase A, 50 μg/mL PI in PBS) on ice for 30 min in the dark. Cell cycle distribution was measured with FACSCalibur flow cytometry using CellQuest software (BD Biosciences, San Jose, CA) and analyzed using WinMDI 2.8 software (J. Trotter, Scripps Research Institute, La Jolla, CA).

### Detection of YO-PRO-1 uptake and nuclear staining with DAPI

For the detection of apoptosis, cells seeded on 60 mm culture dishes were treated with 50 μg/mL of SSE for 6, 12, and 24 h, harvested, and then incubated with apoptosis-specific dye, YO-PRO-1 (Molecular Probes, Eugene, OR) at 10 μM for 5 min. YO-PRO-1 uptake was determined with FACSCalibur flow cytometry using CellQuest software and analyzed using WinMDI 2.8 software. In addition, SSE-treated cells (1 × 10^4^ cells/0.2 mL PBS) were spun onto glass slides by cytospin centrifuge (Cellspin; Hanil, Korea) at 400 × g for 4 min, fixed with 4% paraformaldehyde for 10 min at 37°C, stained with DAPI solution for 10 min, and then observed under the fluorescence microscope (Olympus TH4-200; Olympus Optical Co. LTD).

### Fluorescence analysis of LC3 distribution

Cells (5 × 10^4^) grown on the coverslips in 24-well culture plates were transiently transfected with RFP-LC3 plasmid using TransIT-2020 (Mirus, Madison, WI), treated with 50 μg/mL SSE for 12 h, and then appearance of RFP-LC3 puncta was visualized on a confocal laser scanning microscope (FV10i-W; Olympus Optical Co. LTD) after mounting the coverslips onto glass slides with Vectashield (mounting medium with DAPI, Vector Laboratories, Burlingame, CA).

### Western blot analysis

After washing cells twice with PBS, whole cell lysates were extracted in M-PER Mammalian Protein extraction Reagent (Thermo Scientific, Rockford, IL) by centrifugation (12000 *g* × 15 min, 4°C). Equal amount of protein (20–40 μg) was separated by electrophoresis on 8-15% SDS-polyacrylamide gels, and transferred to Immobilon®-P PVDF transfer membrane (Millipore, Bedford, MA). After immunoblotting, proteins were visualized using a PowerOpti-ECL Western blotting Detection reagent (Animal Gentetics, Inc. Korea) and an ImageQuant LAS 4000 mini (GE Healthcare, Piscataway, NJ). Band intensities were quantified using ImageJ software (National Institutes of Health, USA).

### Preparation of standard and sample

The standard solutions of seven components, puerarin, daidzin, liquiritin, naringin, hesperidin, neohesperidin, and glycyrrhizin were prepared by dissolving 2 mg of each compound in methanol at the concentration to 200 ppm. The SSE powder was dissolved in water at the concentration of 50 mg/mL, and then filtered through a 0.45 μm PVDF membrane filter (ADVANTEC, Japan) before analysis.

### Chromatographic conditions

The experiments were carried out using RP-HPLC-DAD (reverse phase high-performance liquid chromatography-photoiodide array detector) system consisting of a Waters 2695 Alliance (Milford, MA) separation module and a 966 photodiode array detector. The output signal of the detector was recorded using Waters Empower 1.0 software system. The chromatographic separation was conducted with RS-tech C_18_ column (Optomapak C_18_, 4.6 × 250 mm, 5 μm, Daejeon, Korea), the column oven temperature was kept at 40°C, and the injection volume was 20 μL. The wavelength of the UV detector was set at 254 nm and 280 nm. The mobile phase composed of water containing 0.1% trifluoroacetic acid (TFA, A) and acetonitrile (B). The run time was 70 min, the flow rate of the mobile phase was 1.0 mL/min, and the mobile phase program was the gradient elution as follows; 5% B (0–10 min) and 5-15% B (10–15 min), 15-20% B (15–35 min), 20-25% B (35–45 min), and 25-75% B (45–70 min). Chromatographic conditions were summarized in Table 2.

**Table 2:** RP-HPLC-DAD operating conditions for analysis of Samsoeum (SSE)

**Item**	**Condition**	
Instrument	Waters 2695 and 966 photodiode array detector	
Software system	Waters Empower 1.0 software system	
Column	RS tech C_18_ column (5 μm, 4.6 mm × 250 mm)	
Column temperature	40°C	
Injection volume	20 μL	
Mobile phase	Water with 0.1% trifluoroacetic acid (TFA)(A) and Acetonitrile (B)	
Flow rate	1.0 mL/min	
UV wavelength	254 and 280 nm	
**Time (min)**	**0.1% TFA (A)**	**Acetonitrile (B)**
0	95	5
10	95	5
15	85	15
35	80	20
45	75	25
70	25	75

### Statistical analysis

Data are presented as the mean ± S.D. values of at least 3 independent experiments, unless otherwise specified. Statistical significance was analyzed by the two-tailed Student’s *t*-test in Sigma Plot 8.0 software (SPSS Inc., Chicago, IL) and a *P* value of less than 0.05 was considered statistically significant.

## Results and discussion

### SSE treatment induces concentration- and time-dependent cell death and G_2_/M arrest in cancer cells

**Figure 1:**
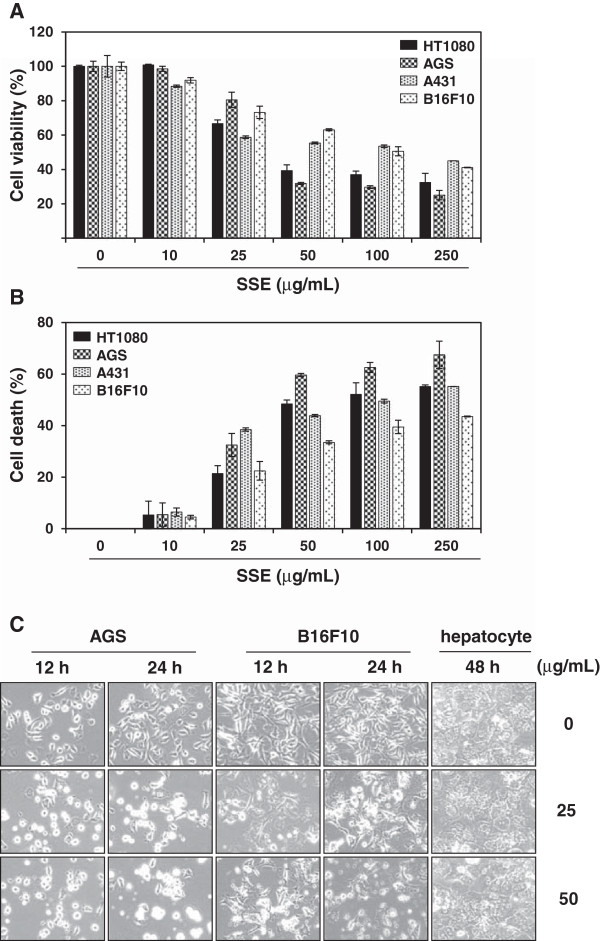
**SSE induces dose- and time-dependent cell death in human and murine cancer cell lines. (A)** Cells seeded on 96-well plates (5 × 10^3^ cells/well) were treated with the indicated concentrations of SSE for 24 h, and the relative cell viability was assessed using MTT assay. **(B)** After incubation of cells with the indicated concentrations of SSE, dead cells were counted using trypan blue exclusion assay. The data represent 3 independent experiments carried out in triplicate and expressed as mean ± SD. **(C)** AGS, B16F10, and murine hepatocytes were incubated with 25 and 50 μg/mL of SSE during the indicated periods and then observed under an inverted microscope.

To investigate the anti-cancer effect of SSE, we treated several human and murine cancer cell lines, including HT1080, AGS, A431, and B16F10, with various concentrations of SSE (10, 25, 50, 100, and 250 μg/mL) for 24 h and assessed cell viability and cell death using MTT assay and trypan blue exclusion assay, respectively. As shown in Figure 1A and 1B, SSE reduced cell viability and caused cell death in proportion to concentration, whereas the relative concentration of DMSO (0.01%) had little influence on cell proliferation (HT1080, IC_50_ = 25.1 μg/mL; AGS, IC_50_ = 42.1 μg/mL; A431, IC_50_ = 344.8 μg/mL; B16F10, IC_50_ = 388.9 μg/mL). Of these cell lines, human gastric carcinoma AGS and murine melanoma B16F10 cell lines were used in all subsequent experiments. Under a phase contrast microscope, viable AGS and B16F10 cells were significantly decreased by SSE treatment in a time- and dose-dependent manner, and the majority of cells shrank and became rounded before detaching from the culture plates (Figure 1C), a typical morphologic appearance in apoptotic cell death. In addition, SSE-treated cancer cells developed a highly granular appearance. Some herbal remedies and dietary supplements have been reported to induce hepatotoxicity because the liver plays an essential role in transforming and clearing chemicals [23]. Therefore, we next examined the effect of SSE on the cell viability of normal hepatocytes. As shown in Figure 1C, normal hepatocytes were unaffected by SSE treatment even after incubation for 48 h at 50 μg/mL, suggesting that SSE is cytotoxic to cancer, but not to normal hepatocytes.

**Figure 2:**
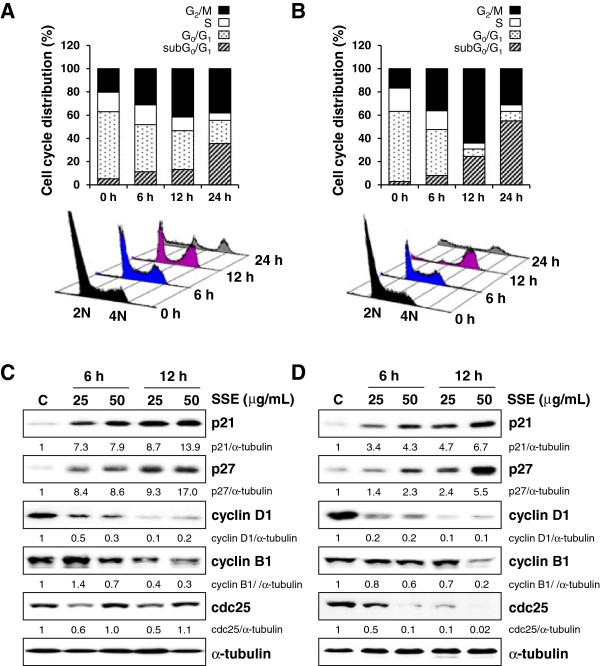
**SSE arrests cell cycle progression at G**_**2**_**/M phase.** AGS **(A)** and B16F10 **(B)** cells were treated with 50 μg/mL SSE for the indicated incubation times (6, 12, and 24 h), fixed with ice-cold 70% ethanol, and stained with propidium iodide solution and then subjected to flow cytometry for the determination of cell cycle distribution. Expression of cell cycle-related proteins was examined with western blot analysis in SSE-treated AGS **(C)** and B16F10 **(D)** cells. The amount of α-tubulin served as the protein loading control, and relative band intensity compared to untreated “control” cells was calculated using ImageJ after normalization to α-tubulin expression. The data represent 3 independent experiments.

For further determination of the potential role of SSE in modulating cell cycle progression, cells were treated with 50 μg/mL SSE for 6, 12, and 24 h, and then the cell cycle distribution was analyzed with PI staining and flow cytometry. In AGS cells, SSE treatment for 6 and 12 h increased the proportion of cells in G_2_/M phase to 31.19% and 41.57%, respectively (Figure 2A) compared with that in untreated cells (20.34%). An increase in cell cycle arrest in G_2_/M phase was also detected in B16F10 cells at 6 and 12 h post-SSE treatment (36.31% and 64.11% versus 16.75%, respectively; Figure 2B), and this increase was accompanied by a corresponding decrease in the proportion of cells in S phase and G_0_/G_1_ phase. Furthermore, 24-h post-SSE treatment, the apoptotic sub-G_0_/G_1_ peak was considerably increased to 35.56% and 55.05% in AGS and B16F10 cells, respectively, indicating that G_2_/M cell cycle arrest by SSE inhibited growth and consequently induced cell death. Consistent with this observation, SSE treatment elevated levels of cyclin-dependent kinase inhibitors p21 and p27 after 6 h of treatment and longer and reduced levels of cyclin D1, cyclin B1, and cdc25 in AGS (Figure 2C) and B16F10 (Figure 2D) cells in a dose- and time-dependent manner compared with those in untreated control cells.

### SSE induces both apoptosis and autophagy in AGS and B16F10 cells

**Figure 3:**
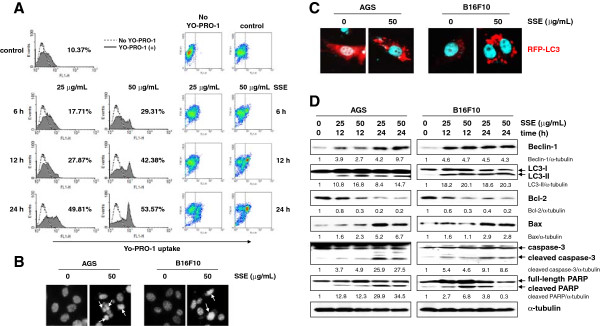
**SSE induces both apoptotic and autophagic cell death. (A)** AGS cells were treated with SSE (25 and 50 μg/mL) for 6, 12, and 24 h, stained with YO-PRO-1, and analyzed using flow cytometry. Histogram and density plot data are shown. **(B)** For the detection of chromatin condensation, cells treated with SSE (50 μg/mL) for 24 h were stained with DAPI and observed under a fluorescent microscope. **(C)** Cells seeded on cover slips were transiently transfected with RFP-tagged LC3 plasmid DNA (RFP-LC3), treated with SSE (50 μg/m) for 12 h, and observed for fluorescence. **(D)** Caspase-3 activation, PARP cleavage, increases in Beclin-1, and LC3-II expression were assessed using western blot analysis after treatment with SSE (25 and 50 μg/mL) for 12 and 24 h. Changes in Bcl-2 and Bax expression after SSE treatment were also determined. Data represent 3 independent experiments.

To analyze whether SSE induces apoptosis or autophagy, we initially assessed the extent of YO-PRO-1 uptake using flow cytometry in AGS cells undergoing SSE-induced cell death. Permeability to YO-PRO-1 is an early event in apoptotic cell death and occurs well before the loss of membrane integrity [24]. Accordingly, YO-PRO-1 uptake was considerably increased to 17.71% and 29.31% even after 6 h treatment at concentrations of 25 and 50 μg/mL, respectively, compared with that of control cells (10.37%), and further accumulation occurred in proportion to incubation time and concentration (Figure 3A). SSE treatment for 24 h at 50 μg/mL resulted in an approximately 5.2-fold (53.57%) increase in the apoptotic rate (10.37% for control). After DAPI staining, AGS and B16F10 cells treated with SSE (50 μg/mL) for 24 h exhibited chromatin condensation (Figure 3B, white arrows). Next, to determine whether SSE induces autophagy, we examined the intracellular distribution of LC3, an autophagy marker, in response to SSE treatment in AGS and B16F10 cells transfected with an expression construct for LC3 fused to red fluorescent protein (RFP-LC3) under a confocal microscope. As shown in Figure 3C, in AGS cells, RFP-LC3 was evenly diffused throughout the cytoplasm in control cells, whereas SSE-treated cells displayed a punctuate pattern of RFP-LC3 fluorescence, indicating the association of RFP-LC3 with the autophagosomal membrane. In B16F10 cells, SSE treatment remarkably increased punctuate pattern of RFP-LC3 fluorescence. LC3, the mammalian equivalent of yeast Atg8, is cleaved from LC3-I (18 kDa) to LC3-II (16 kDa) during autophagy via proteolytic cleavage and lipidation, and this modification of LC3 is essential for the formation of autophagosomes and completion of autophagy [25]. LC3-I and LC3-II are localized in the cytosol or in autophagosomal membranes, respectively; thus, the redistribution of LC3 in autophagosomal membranes as observed in Figure 3C may be strong evidence for autophagy induction.

To gain further insight into the mechanism by which SSE induces cell death, we examined the effect of SSE treatment on the expression of apoptosis- and autophagy-related proteins using western blot analysis (Figure 3D). The protein levels of Beclin-1, which initiates autophagosome formation during autophagy, were gradually increased in AGS and B16F10 cells after SSE treatment. Moreover, the ratio of LC3-II to LC3-I was significantly increased in SSE-treated AGS and B16F10 cells. In addition, SSE treatment significantly inhibited anti-apoptotic *Bcl-2* expression, enhanced pro-apoptotic *Bax* expression, and resulted in the cleavage of caspase-3 and PARP, a downstream target of activated caspase-3. Bcl-2 family proteins including Bcl-2 and Bcl-xL are frequently overexpressed in cancers and inhibit apoptosis by binding to Bax or Bak [26]. Moreover, Bcl-2 and Bcl-xL suppress autophagy by binding to the BH3 domain (amino acids 114–223) of the Beclin-1 protein and sequestering Beclin-1 from hVps34, which is a significant regulator in the initial steps of autophagy, indicating that Bcl-2 and Bcl-xL play essential roles in the crosstalk between autophagy and apoptosis [27]. These data suggest that SSE treatment efficiently induces both autophagy and apoptosis, which partner to induce cell death cooperatively by modifying Beclin-1 and Bcl-2 expression.

### SSE suppresses the PI3K/Akt/mTOR pathway via activation of AMPK and activates the mitogen-activated protein kinase (MAPK) pathway

**Figure 4:**
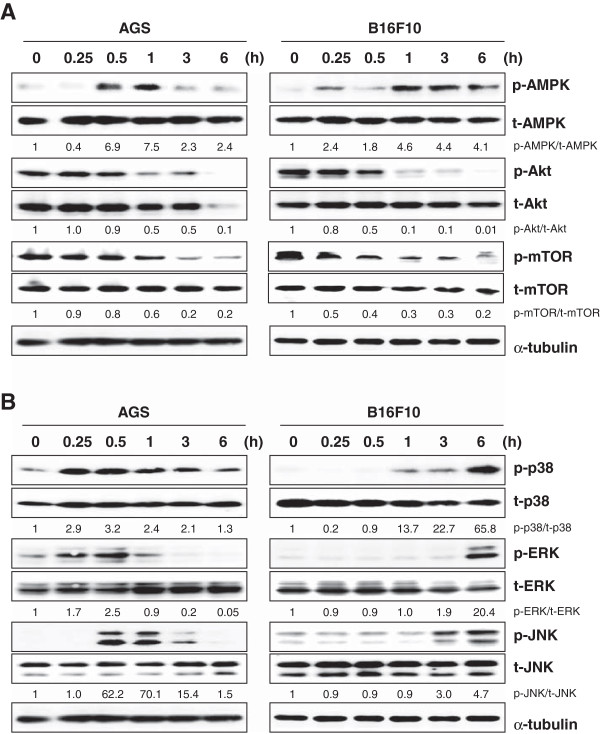
**SSE inhibits Akt/mTOR pathway and activates MAPKs pathway. (A)** Cell lysates were prepared after treatment with SSE at 50 μg/mL for 0.25, 0.5, 1, 3, and 6 h, and then subjected to western blot analysis for the determination of levels of AMPK, Akt, mTOR, and their phosphorylated forms. **(B)** Lysates were also determined for the levels of p38, ERK, JNK, and phosphorylated forms. Data represent 2 independent experiments.

The PI3K/Akt/mTOR signaling pathway is frequently activated in human cancers, and it modulates cancer cell proliferation, metastasis, and acquired drug resistance. Activation of class I PI3K inhibits apoptosis and autophagy through activation of Akt and mTOR [28]. Beclin-1 expression and Akt/mTOR pathway inhibition are consistently connected with the induction of autophagy in cancer cells [27,29]. Previous studies have demonstrated that autophagy is regulated by multiple signaling pathways, including class III PI3K, class I PI3K/Akt/mTOR, and MAPKs. To determine whether SSE-induced cell death involves the PI3K/Akt/mTOR signaling pathway, we measured the phosphorylation status of Akt at Ser473, mTOR at Ser2481, and AMPK, a repressor of mTOR, at Thr172 in SSE-treated AGS and B16F10 cells using western blot analysis. As shown in Figure 4A, treatment of AGS and B16F10 cells with 50 μg/mL SSE significantly increased AMPK phosphorylation and reduced Akt and mTOR phosphorylation. A recent study has shown that JNK activation during nutrient starvation induces Bcl-2 phosphorylation and Beclin-1 expression, eventually promoting apoptosis and autophagy by dissociating Bcl-2 from Bax and disrupting the Bcl-2-Beclin-1 complex, respectively [30]. Additionally, sustained activation of mitogen-activated protein kinase/extracellular signal-regulated kinase (ERK) downstream of AMPK reportedly leads to a marked increase in Beclin-1 expression [31], and ER stress-induced Beclin-1 expression and autophagy induction correlate with increased p38 activation. In our study, SSE treatment significantly increased phosphorylation of p38, ERK, and JNK (Figure 4B). In AGS cells, MAPK phosphorylation peaked 30 min after SSE treatment, whereas this peak was reached at 6 h in B16F10 cells. Taken together, these results demonstrate that SSE induces cell death by inhibiting Akt and mTOR activity and by activating the MAPK pathway.

### JNK activation is required for the up-regulation of Beclin-1, LC3-II, and Bax and down-regulation of Bcl-2 expression in response to SSE

**Figure 5:**
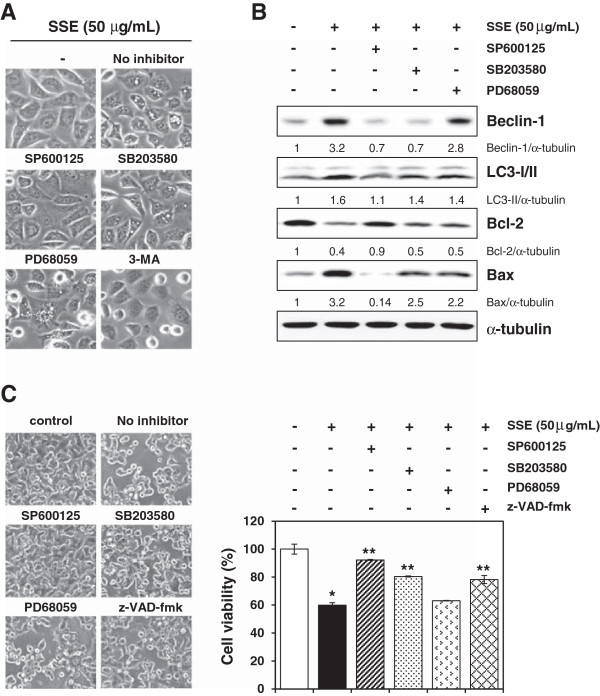
**JNK activation is required for the up-regulation of Beclin-1 and subsequent cell death by SSE. (A)** After pre-incubation with specific inhibitors including SP600125 (10 μM), SB203580 (10 μM), PD98059 (10 μM), or 3-MA (100 μM) for 1 h, B16F10 cells were treated with SSE at 50 μg/mL for 24 h and then observed under phase contrast microscope. **(B)** The effect of specific MAPK inhibitors on the expression of Beclin-1, LC3-I/-II, Bcl-2, and Bax was evaluated. Cell lysates were prepared after incubation for 12 h with specific inhibitors for 1 h before SSE treatment. **(C)** After pre-incubation with or without specific inhibitors, cells were treated with 50 μg/mL SSE for 24 h, and cell viability was determined using MTT assay. Data acquired from 2 independent experiments are expressed as mean ± SD. *p < 0.05 versus control, **p < 0.05 versus SSE-treated cells.

To investigate further the role of MAPK activation in SSE-mediated cell death, we pre-incubated cells with or without pharmacological inhibitors of JNK (SP600125, 10 μM), p38 (SB203580, 10 μM), or ERK (PD98059, 10 μM) for 1 h, followed by SSE treatment for 24 h. As shown in Figure 5A, cells treated with SSE showed morphological features of cytoplasmic vacuole accumulation and only pre-incubation with SP600125 nearly blocked vacuole formation in a manner similar to 3-MA, an inhibitor for autophagosome formation. Immunoblot analysis showed that pre-incubation with SP600125 completely prevented the induction of Beclin-1, LC3-II, and Bax and reduction of Bcl-2 by SSE treatment to the extent observed in untreated control cells, whereas pre-incubation with SB203580 and PD98059 showed partial or few inhibitory effects compared to that of SP600125 (Figure 5B). SP600125 also significantly protected SSE-treated cells from cell death by about 80%, whereas SB203580 showed a partial effect of approximately 50%, and PD98059 had little effect (Figure 5C). Moreover, pre-incubation with z-VAD-fmk, a pan-caspase inhibitor, showed a partial inhibitory effect. Collectively, these data indicate that SSE-mediated cell death is mainly contributed by JNK activation, followed by modification of autophagy- and apoptosis-related protein expression.

### Identification of seven main components in SSE by RP-HPLC-DAD system

**Figure 6:**
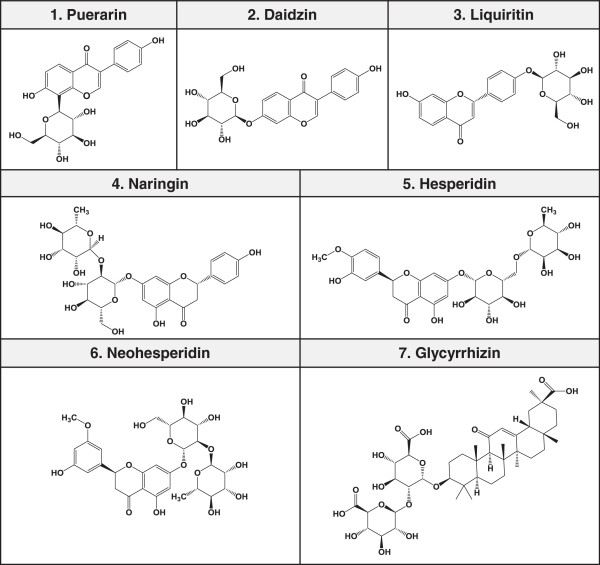
Chemical structures of 7 standard compounds in SSE.

**Figure 7:**
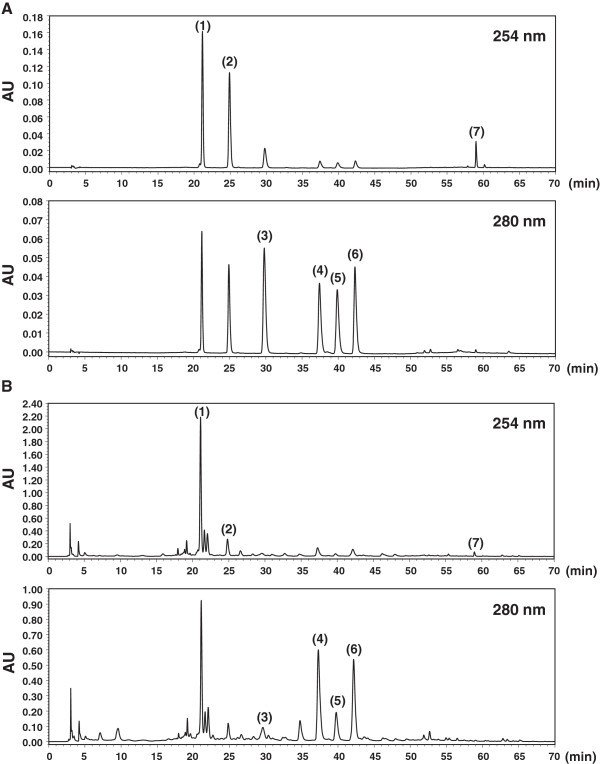
**RP-HPLC-DAD chromatograms of 7 major compounds.** puerarin (1), daidzin (2), liquiritin (3), naringin (4), hesperidin (5), neohesperidin (6), and glycyrrhizin (7) in the standard mixture **(A)** and SSE sample **(B)** identified at wavelengths of 254 and 280 nm.

HPLC analysis was performed for the identification of 7 main components in SSE, including puerarin, daidzin, liquiritin, naringin, hesperidin, neohesperidin, and glycyrrhizin (Figure 6). A C_18_ column was used for analysis, and the flow rate of the mobile phase was fixed at 1.0 mL/min. To achieve the desired separation, we tested the gradient elution proportions of water and acetonitrile. Trifluoroacetic acid was added to water to advance a peak shape and inhibit peak tailing. The ultraviolet (UV) wavelength of the 7 components was adjusted based on the maximum UV spectra absorption of each component. As shown in Figure 7A, puerarin, daidzin, and glycyrrhizin were detected at 254 nm, and liquiritin, naringin, hesperidin, and neohesperidin were detected at 280 nm. Each component in SSE was characterized by comparing retention time and UV spectra. The profiles of puerarin (1, *t*_*R*_: 21.10 min), daidzin (2, *t*_*R*_: 24.82 min), liquiritin (3, *t*_*R*_: 29.62 min), naringin (4, *t*_*R*_: 37.28 min), hesperidin (5, *t*_*R*_: 39.75 min), neohesperidin (6, *t*_*R*_: 42.17 min), and glycyrrhizin (7, *t*_*R*_: 58.97 min) were identified in the SSE samples (Figure 7B). A previous study has reported that hesperidin decreases Bcl-2 expression and increases Bax and active caspase-3 expression, resulting in apoptotic cell death in human colon cancer cells [32]. Puerarin has an apoptotic effect in colon cancer via caspase-3 activation and Bcl-2 down-regulation [33]. A recent study has reported that naringin induces Fas death receptor- and mitochondria-mediated apoptosis in human cervical carcinoma and SiHa cells [34] and causes G_1_ cell cycle arrest via up-regulation of p21 via activation of the Ras/Raf/ERK pathway in urinary bladder carcinoma 5637 cells [35]. Other reports have demonstrated that glycyrrhizin exhibits potent cytotoxic effects against human prostate cancer cells LNCaP and DU145 via caspase-3 and caspase-8 activation [36]. These facts suggest that the anti-cancer activity of SSE might be related to these active components.

**Figure 8:**
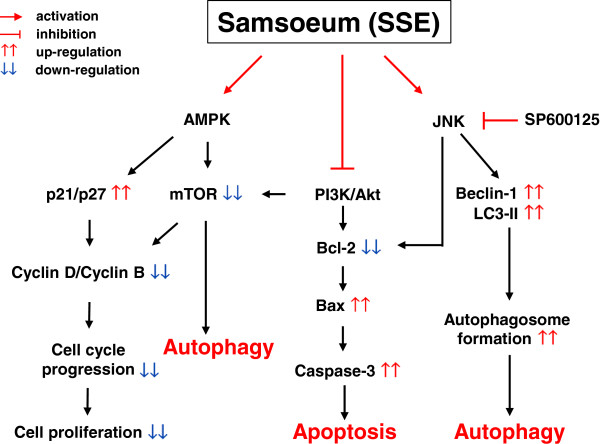
Schematic diagrams showing the mechanisms underlying anti-cancer effects of SSE.

Because advanced malignancies require treatment targeting multiple cellular pathways, properly formulated herbal cocktails are believed to take advantage of synergy and achieve better therapeutic efficacy than that of a single herb. In the present study, we found that both apoptosis and autophagy contribute to cancer cell death induced by SSE, a traditional herbal formula, in a complementary and cooperative fashion by regulating key signaling pathways upstream of these 2 processes. Our data revealed that SSE efficiently inhibits the growth of and causes death in several cancer cell lines, including AGS and B16F10 cells, which are commonly associated with G_2_/M cell cycle arrest, regulation of anti-apoptotic and pro-apoptotic proteins, activation of caspase-3, increase of Beclin-1, and conversion of LC3-I to LC3-II. Disruption of the PI3/Akt pathway culminating in Akt inactivation reportedly plays a critical role in apoptosis as well as autophagy induction. Additionally, Bcl-2 regulates PI3K/Akt signaling, in turn positively regulating the mTOR signaling pathway, which can inhibit autophagy induction; it also down-regulates Beclin-1-dependent autophagy by interacting with Beclin-1 or by inhibiting the formation of the Beclin-1-hVps34 PI3K complex, which can enhance autophagy (Figure 8) [17,27]. The relationship between these types of PCD is still controversial because they may cooperate, coexist, or antagonize each other to balance survival with death. However, our experiments using pharmacological inhibitors demonstrated that SSE treatment displayed collaborative interplay between apoptosis and autophagy for cell death.

## Conclusions

In summary, our finding clearly demonstrated that SSE has anti-cancer activity via suppression of the Akt/mTOR signaling pathway through AMPK activation, which resulted in the down-regulation of Bcl-2 and up-regulation of Beclin-1. SSE treatment activated MAPKs including p38, ERK, and JNK; however, only SP600125, a specific inhibitor for JNK activation, nearly prevented SSE-induced cell death by blocking changes in the level of key proteins such as Bcl-2, Bax, Beclin-1, and LC3-II. In particular, SSE causes both apoptosis and autophagy, and these PCD processes are indispensable for the induction of cell death. Collectively, these results provide new insight into the pharmacological action of SSE as a potent herbal medicine for the treatment of malignant tumors.

## Abbreviations

SSE: Samsoeum; PCD: Programmed cell death; mTOR: Mammalian target of rapamycin; PI3K: Phosphatidylinositol 3 kinase; Bcl-2: B-cell lymphoma 2; AMPK: Adenosine monophosphate activated-activated protein kinase; LC3: Microtubule-associated protein light chain 3; 3-MA: 3-methyladenine; RP-HPLC-DAD: Reverse phase high-performance liquid chromatography-photoiodide array detector.

## Competing interests

All authors are in agreement with the content of the manuscript and declare no financial or intellectual conflicts of interests regarding this study.

## Authors’ contributions

AYK conceived of the study, carried out experiments including PI staining, YO-PRO-1 uptake, RFP-LC3 redistribution, and western blotting, and drafted manuscript. NHY participated in cell culture, determination of cytotoxicity, preparation of SSE and HPLC analysis. JYM participated in the design and coordination of study. All authors read and approved the final manuscript.

## Pre-publication history

The pre-publication history for this paper can be accessed here:

http://www.biomedcentral.com/1472-6882/13/233/prepub
